# Comparative electron microscopy particle sizing of TiO_2_ pigments: sample preparation and measurement

**DOI:** 10.3762/bjnano.15.29

**Published:** 2024-03-25

**Authors:** Ralf Theissmann, Christopher Drury, Markus Rohe, Thomas Koch, Jochen Winkler, Petr Pikal

**Affiliations:** 1 Research Services, KRONOS INT. Inc., Peschstr. 5, 51737 Leverkusen, Germany; 2 TRONOX Pigment UK Ltd., Laporte Road, Stallingborough, North East Lincolnshire, DN40 2PR, United Kingdom; 3 Global Special Analytics, Venator Germany GmbH, Dr.-Rudolf-Sachtleben-Str. 4, 47198 Duisburg, Germany; 4 Billions Europe Ltd. (LB Group), Winder House, Kingfisher Way, Stockton on Tees, TS18 3EX, United Kingdom; 5 R&D, PRECHEZA, nábř. Dr. Edvarda Beneše 1170/24, 750 02 Přerov, Czech Republic

**Keywords:** electron microscopy, nanomaterials definition, number-based, particle sizing, primary particles

## Abstract

Titanium dioxide (TiO_2_) pigment is a non-toxic, particulate material in widespread use and found in everyone’s daily life. The particle size of the anatase or rutile crystals are optimised to produce a pigment that provides the best possible whiteness and opacity. The average particle size is intentionally much larger than the 100 nm boundary of the EU nanomaterial definition, but the TiO_2_ pigment manufacturing processes results in a finite nanoscale content fraction. This optically inefficient nanoscale fraction needs to be quantified in line with EU regulations. In this paper, we describe the measurement procedures used for product quality assurance by three TiO_2_ manufacturing companies and present number-based primary particle size distributions (PSDs) obtained in a round-robin study performed on five anatase pigments fabricated by means of sulfate processes in different plants and commonly used worldwide in food, feed, pharmaceutical and cosmetic applications. The PSDs measured by the three titanium dioxide manufacturers based on electron micrographs are in excellent agreement with one another but differ significantly from those published elsewhere. Importantly, in some cases, the PSDs result in a different regulatory classification for some of the samples tested. The electron microscopy results published here are supported by results from other complementary methods including surface area measurements. It is the intention of this publication to contribute to an ongoing discussion on size measurements of TiO_2_ pigments and other particulate materials and advance the development of widely acceptable, precise, and reproducible measurement protocols for measuring the number-based PSDs of particulate products in the size range of TiO_2_ pigments.

## Introduction

Following the EU definition of nanomaterials as being materials in which more than 50% by number of their primary particles have at least one dimension smaller than 100 nm [1–2], the accurate measurement of particle size distributions (PSDs) has become critical. Among the methods used to determine particle size distributions, the evaluation of particle sizes from electron microscopy (EM) images is considered as a confirmatory method for correct classification [3]. In the case of monomodal particle distributions, this method is straightforward [4]. However, when the PSD is broad, the sample preparation plays a crucial role in obtaining reproducible and unbiased results [5–6].

The standard approach to measuring particle size by EM is to thoroughly disperse the particles and then to evaluate them using an automatic or semi-automatic procedure. This method is effective for loosely bound monodisperse particles such as polystyrene latex or gold particles. However, it can be challenging when dealing with highly agglomerated and cohesive particles such as TiO_2_, especially for laboratories without prior experience. The authors have chosen to use “E171” as an abbreviation for the former “European food pigment E171”. The use of TiO_2_ E171 for food application was banned by the European Commission in January 2022 based on a European Food Safety Authority (EFSA) evaluation that a “concern for genotoxicity of TiO_2_ particles that may be present in E 171 could not be ruled out” [7]. Recently, papers have been published in which a number of E171 samples were found by transmission electron microscopy (TEM) to be nanomaterials according to the EU definition [8–9]. For TiO_2_ producers this is surprising as the former food-grade titanium dioxide E171 is manufactured as a standard white pigment grade, which would be significantly inferior if more than 50% of its particles were below 100 nm in diameter. In other papers, samples of the same materials were measured using scanning electron microscopy (SEM) and found not to meet the EU classification for nanomaterials [10–11]. In 2018, KRONOS INT. Inc., Precheza a.s and Venator supported EFSA in responding to the questions raised by the EFSA Scientific Panel on the particle size distribution of E171. The results were reported by EFSA in 2019 [11]. The same samples as measured in [8] and [11] were used in a round-robin test in 2023 by three laboratories (KRONOS INT. Inc., Precheza a.s, and Venator). The results are summarised in this publication. Each laboratory followed its own routine procedure to prepare samples, make SEM images, and measure several particle size parameters including the smallest dimension (MinFeret), largest dimension (MaxFeret), and equivalent circular diameter (ECD). Results from the “Regional Centre of Advanced Technologies and Materials”, Olomouc, Czech Republic (RCPTM) were also included.

It is good practice to compare the PSD estimated by EM with other methods for determination and validation. For untreated, non-porous materials, the specific surface area (SSA) serves as a useful independent method. The SSA of the pigment samples was calculated from the particle size distributions, and these values were directly compared with the measured SSA. As with all titanium dioxide materials, the optical properties of E171 are related to the primary particle size, although the degree of dispersion needs to be taken into account [12].

An overview of particle size measurement methods for nanomaterials is given in [4]. Our paper focuses mainly on number-based particle size measurements for E171 using EM investigations and the comparison with optical properties, SSA, and single-particle inductively coupled mass spectrometry (spICP-MS) to accompany the reported EM results. An overview covering industrially applied measurement methods for pigments and fillers is given in the JRC Technical Report [13]; EM particle size measurements are covered in OECD guidelines [14] as well as in ISO standards [ISO/TS 19749 (published 07/21) and ISO/TS 21363:2020 (published 11/21)].

The key to any visual EM particle sizing method is to take random images of the sample, to evaluate all measurable particles on each image, and to correctly identify the particle edges. Effective image acquisition and analysis requires training and routine performance checks for both the personnel capturing the images and those interpreting them.

## Results

### Electron microscopy measurements of E171 samples and related calculations

An initial estimate of the particle size range of the pigments under investigation was calculated by assuming a log-normal PSD around a median (μ*) of 100 nm and a distribution width (σ*) of 1.65 nm. In this “worst case” estimate, 99% of the particles have diameters in the range between 22 and 445 nm, setting the requirements for the measurement conditions. These must allow the smallest particles to be imaged with sufficient resolution and record a field of view such that it does not cut off a significant fraction of the largest particles. With a resolution of 1.4 nm/pixel and a field of view of 1800 nm × 1250 nm (corresponding to measurement conditions M2), particles as small as a diameter of approx. 14 nm can be measured. Only particles that are completely within the field of view can be measured. The probability that a particle of a given size satisfies this geometric constraint is calculated, and the result is given in Table 1. The larger particles are therefore systematically underrepresented in any EM measurement.

**Table 1:** Probability that a particle of a given size will fall completely within the 1800 nm × 1250 nm field of view.

Particle size (nm)	50	100	200	400

Probability (%)	93.3	86.9	74.7	52.9

As such, any microscopic measurement is biased towards smaller particles because of simple geometric constraints; the higher the resolution and the smaller the field of view, the more pronounced this becomes.

For EM measurements, all five samples were prepared and measured by the participating companies according to their own standard operating procedures as described in the Experimental section. The evaluation procedures and sample preparations used here have been developed over many years and are regularly used in each participating company for quality control and research purposes.

**Figure 1:**
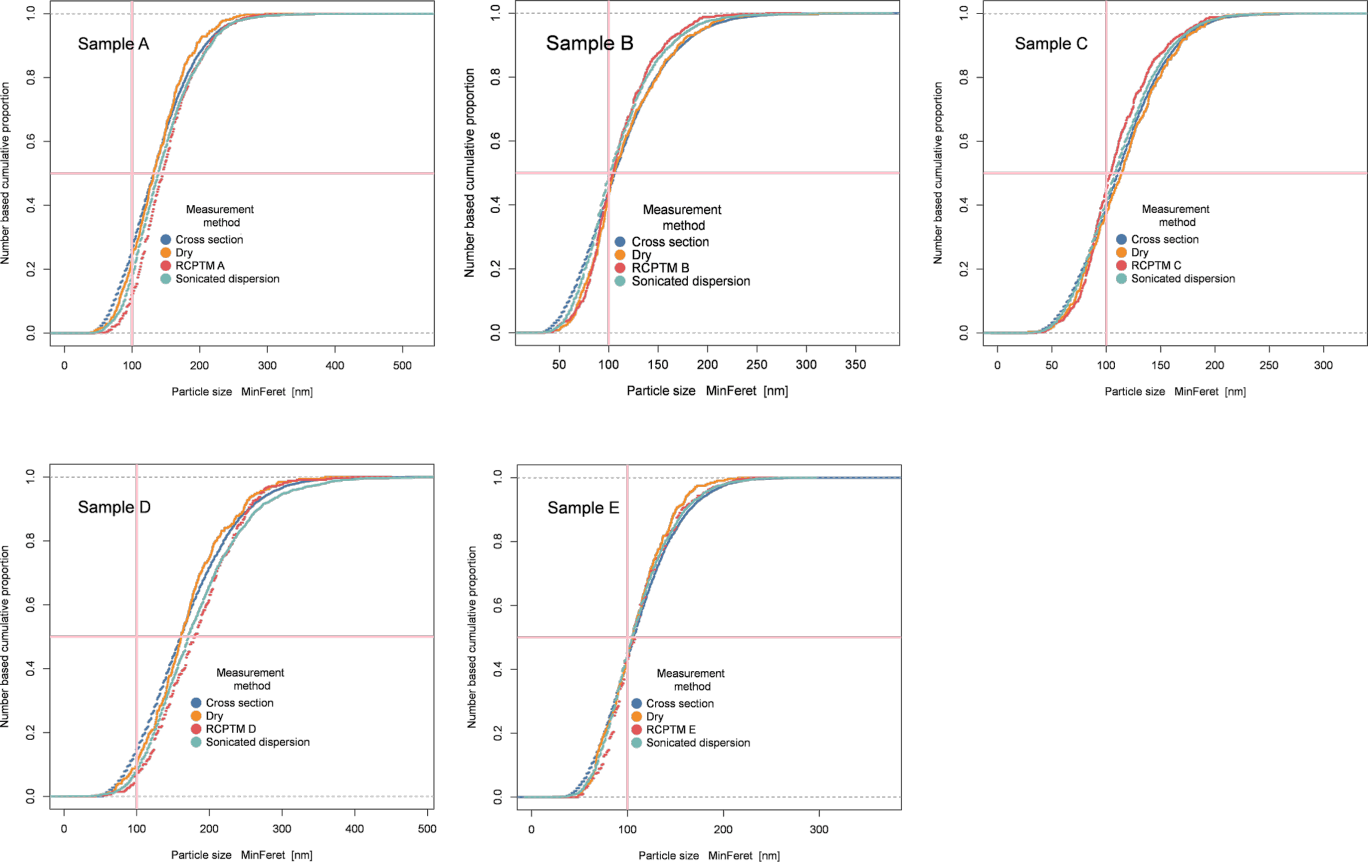
Comparison of cumulative distribution curves measured by three different manufacturers using three different methods (M1: cross section, M2: dry, and M3: sonicated dispersion) and by the external laboratory RCPTM.

The evaluated MinFeret and ECD values, with the exception of M3 for sample D, fall within the 95% confidence interval (Table 2). The observed standard deviation of the fraction of particles smaller than 100 nm is less than 5% for each sample. The cumulative distribution curves obtained by the three different preparation, measurement, and evaluation methods are shown in Figure 1. Excellent agreement is observed for samples A–C and E. Sample D, with the largest particle size and lowest nanoscale fraction shows the largest deviations, but even here the reproducibility is good.

**Table 2:** Results from three different electron microscopy particle size measurement methods of five anatase E171 samples.

E171	MinFeret number-based (measured)		MinFeret number-based (fitted)		KS test for lognormal distribution	Aspect ratio	<100 nm MinFeret	<100 nm MinFeret	Meas. particles	Meas. method

	D50_n_	SE^a^ (95%)	μ*	σ*	*p*		Meas.	Fitted		
	nm	nm	nm	nm	%		%	%	*N*	

A	132	1	127	1.5	4.0	1.16	26	27	6759	M1
	132	5	128	1.4	5.2	1.19	24	23	366	M2
	139	2	137	1.4	2.4	1.2	18	18	2065	M3
B	107	1	103	1.4	2.8	1.16	44	46	7754	M1
	105	4	108	1.4	4.5	1.2	44	40	394	M2
	102	2	102	1.4	2.6	1.21	49	47	1608	M3
C	110	1	106	1.4	4.6	1.16	40	43	8118	M1
	114	5	109	1.4	5.2	1.2	37	40	313	M2
	108	1	105	1.4	3.7	1.21	41	44	4420	M3
D	161	2	155	1.5	3.9	1.15	15	13	6941	M1
	161	7	157	1.4	5.5	1.21	10	10	320	M2
	171	3	168	1.5	3.0	1.21	8	9	1783	M3
E	107	1	104	1.4	3.3	1.15	43	46	8441	M1
	106	4	102	1.4	4.6	1.19	44	46	313	M2
	104	2	104	1.4	1.8	1.21	45	45	2271	M3

^a^SE standard error of the median based on [15].

The data were fitted under the assumption that the particle size has a log-normal distribution, the values μ* and σ* were calculated, and the Kolmogorov–Smirnov (KS) test was applied to test the significance of the log-normal assumption. The probability *p* that the particle size distribution is not log-normal is given in Table 2 together with the results of the fits.

### Indirect particle size measurements and optical properties

Single-particle inductively coupled plasma mass spectrometry (spICP-MS) measurements are often used to complement EM particle counting results for particulate products with a substantial particle content below 100 nm [16–17]. Therefore, spICP-MS experiments are also included here for comparison. The experiments were performed on an Agilent 8900 triple quadrupole system at Agilent’s application laboratory in Waldbronn, Germany, and the particle counting threshold was set at 30 nm. The samples were first intensively dispersed by ultrasonication in a polyphosphate solution in water (562 J/mL, 2.5% pigment, 2% polyphosphate) one day before measurement. The energy of 562 J/mL was chosen because aggregates and primary particles start to be destroyed above 250 J/mL, but only at low rates [18–19]. However, dispersion energies of approximately 600 J/mL or higher may significantly break aggregates into primary particles, causing a shift in the particle size distribution towards a smaller median size (D50_n_) due to the detection of a higher number of liberated primary particles.

After dispersion, the samples were transported to the Agilent laboratory. The following day, they were re-dispersed in two different ways just before measurement. This was done to evaluate the impact of sample preparation on the results and confirm the method’s suitability for TiO_2_ pigment analysis. One sample was manually agitated, while the other underwent a 1 min dispersion in an 80 W ultrasonic bath.

**Figure 2:**
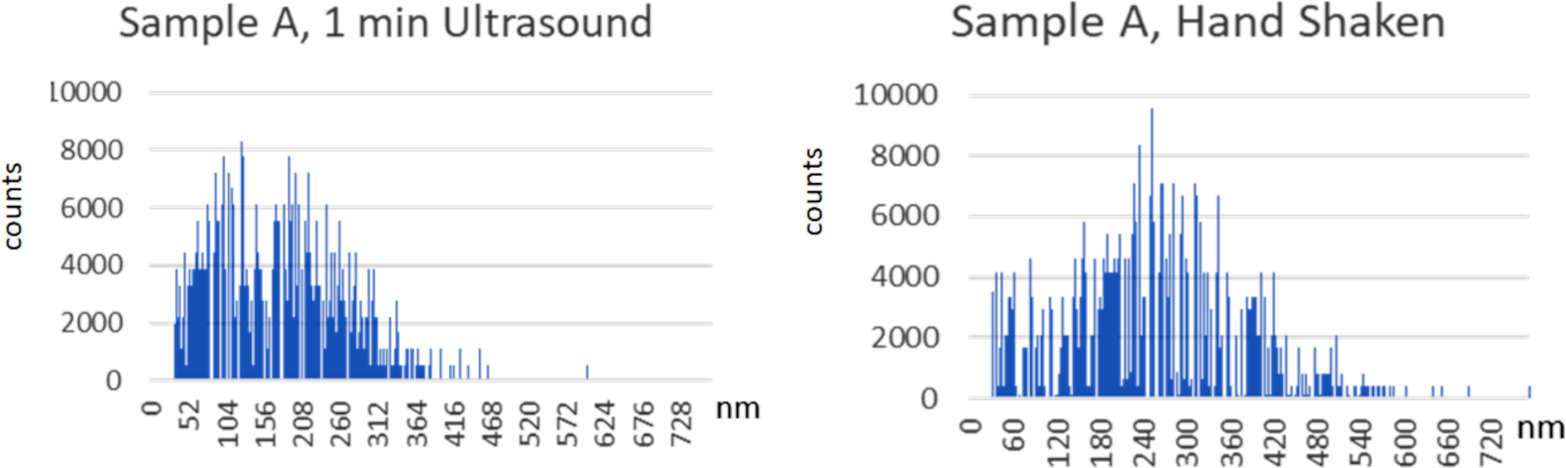
spICP-MS of pre-dispersed (562 J/mL ultrasonic treatment prepared one day before measurement) and stabilised (2% polyphosphate) suspension. Left: intensive re-dispersion for 1 min in an ultrasonic bath at 80 W, right: light re-dispersion through shaking by hand. Both particle size distributions show two maxima, one for primary particles and one for aggregates and agglomerates. Re-dispersion destroys agglomerates that formed overnight and increases the number of primary particles.

For sample A, the bimodal PSD typical of E171 is clearly visible in both spectra in Figure 2. Similar bimodal results were obtained for the other samples. The first peak corresponds to the primary particles and the second peak to the aggregates and agglomerates of the primary particles. The percentage of agglomerates should be higher if shaking by hand is the only re-dispersion step, and this is indeed observed.

As expected, some agglomerates and aggregates persist even after thorough dispersion. In both distributions in Figure 2, the number of particles above 500 nm is low. This is surprising as large agglomerates above 600 nm are typical for hand-shaken dispersions but are not detected here by spICP-MS.

Table 3 shows the measured spICP-MS median particle sizes for the five E171 pigments including results for pure solvents. All medians are above 100 nm. The estimated particle size (D50_n_ - ECD, see Experimental section) is significantly higher than the EM measurement results because of the unavoidable presence of undispersed aggregates and agglomerates.

**Table 3:** spICP-MS results for samples A–E.

	Nebulisation efficiency	Particle concentration (particles/L)	Median size (nm)

dispersion liquid	0.045	3.40E+05	281
sample A	0.045	1.50E+08	150
sample B	0.045	2.40E+08	137
sample C	0.045	2.20E+08	180
sample D	0.045	2.20E+08	249
sample E	0.045	2.70E+08	208

The SSA and light scattering properties of the five samples were examined. To allow for a broader correlation of size parameters with SSA and CIELAB coordinates, an additional set of laboratory samples with a wider range of particle sizes was prepared and evaluated (see Experimental section). The EM MinFeret and EM ECD values according to measurement method M2 are given in Table 4, together with the SSA, the calculated specific surface area (c-SSA), and the CIELAB L*, a*, and b* coordinates showing the light scattering properties of each sample.

**Table 4:** Comparison of optical properties, specific surface area, spICP-MS, and particle size measured by EM for additional laboratory samples and E171 pigments A–E.

Sample	CIE L*	CIE a*	CIE b*	density^a^	c-SSA^b^	SSA	MinFeret	ECD (M2)	spICP-MS

					calculated	measured	D50_n_	D50_n_	D50_n_
				g/cm^3^	m^2^/cm^3^	m^2^/g	nm	nm	nm

Lab 1	46.3	0.14	−1.40	3.9	–	19.7	46	50	–
Lab 2	48.1	0.01	−1.25	3.9	–	17.0	61	67	–
Lab 3	49.4	0.13	−0.99	3.9	–	14.3	61	66	–
Lab 4	49.9	0.13	−1.02	3.9	–	14.1	80	88	–
Lab 5	51.0	0.07	−0.79	3.9	–	12.0	93	101	–
Lab 6	51.3	0.07	−0.68	3.9	–	12.1	105	111	–
Lab 7	51.7	0.03	−0.39	3.9	–	11.2	106	117	–
Lab 8	52.7	−0.03	0.99	3.9	–	7.8	156	173	–
Lab 9	51.6	0.16	2.99	3.9	–	6.1	213	237	–
E171-A	52.4	−0.03	0.16	3.887	10.3	8.5	132	142	150
E171-B	52.5	−0.02	−0.34	3.889	12.9	10.1	105	116	137
E171-C	52.6	0.02	−0.51	3.879	12.4	10.1	114	124	180
E171-D	52.9	0.04	1.91	3.921	8.6	6.9	161	178	249
E171-E	51.6	0	−0.64	3.892	13.1	10.2	106	113	208

^a^The density of Lab samples is an approximation based on results from E171 A–E samples and the known tabulated density range for anatase. ^b^c-SSA was calculated using only the average ECD values.

The complete EM particle size distributions were used to calculate the SSA values for the five samples using three distinct models, namely one based on particle ECD, another on MinFeret values, and the third on the Cauchy model. These results are summarised in Table 5.

**Table 5:** Measured SSA and SSA calculated by various models from the entire particle size distributions.

Sample	Particle size measurement method	Specific surface area (m^2^/g)				density

		calculated from				g/cm^3^
		Cauchy^a^	ECD	MinFeret	measured^b^	

A	M1	8.04	7.86	8.7	8.5	3.887
	M2	8.29	8.43	9.33		
	M3	7.56	7.73	8.51		
B	M1	9.56	9.36	10.35	10.1	3.889
	M2	9.44	9.61	10.61		
	M3	9.8	10.02	11.04		
C	M1	10.18	9.97	11.04	10.1	3.879
	M2	9.86	10.01	11		
	M3	10.05	10.27	11.31		
D	M1	6.6	6.45	7.12	6.9	3.921
	M2	6.65	6.78	7.55		
	M3	5.85	6	6.61		
E	M1	10.19	10.01	11.03	10.2	3.892
	M2	10.66	10.91	12.09		
	M3	10.16	10.37	11.36		

^c^Calculated using average values of MinFeret and MaxFeret; ^b^measured by Precheza, average of four measurements.

Comparing the calculated SSA values in Table 4 and Table 5, it is clear that using only average ECD values for calculating the SSA results in significantly higher values. To obtain accurate SSA estimates from EM measurements, it is essential to utilise the entire measured distribution curve in the calculation process.

## Discussion

Five different electron microscopy (EM) techniques and sample preparations, three reported here and two reported by Verleysen et al. [8], were applied to the same five E171 anatase samples A–E. We also include average values from the EFSA report [11] and results from an external laboratory (RCPTM) for completeness. The results in [11] were obtained by the same laboratories using the same, or slightly adopted, measurement protocols as in this report. To check the stability of samples and the consistency of the measurement protocols used in the participating laboratories over time, we re-measured these five samples in 2023.

**Figure 3:**
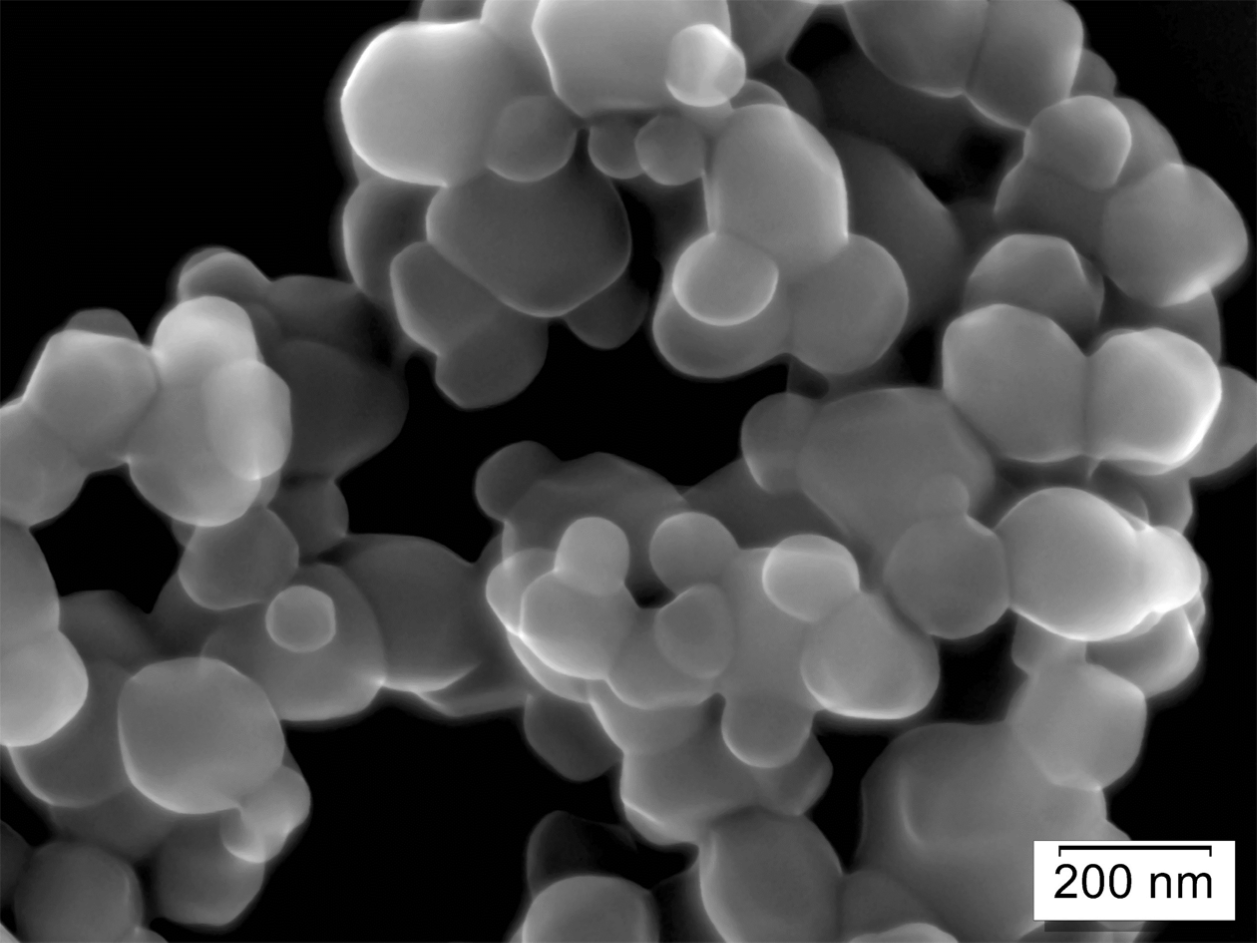
SEM top-view image; in-lens SE detector, resolution: 0.99 nm/pixel, field of view (full image): 2.5 μm × 1.9 μm.

**Figure 4:**
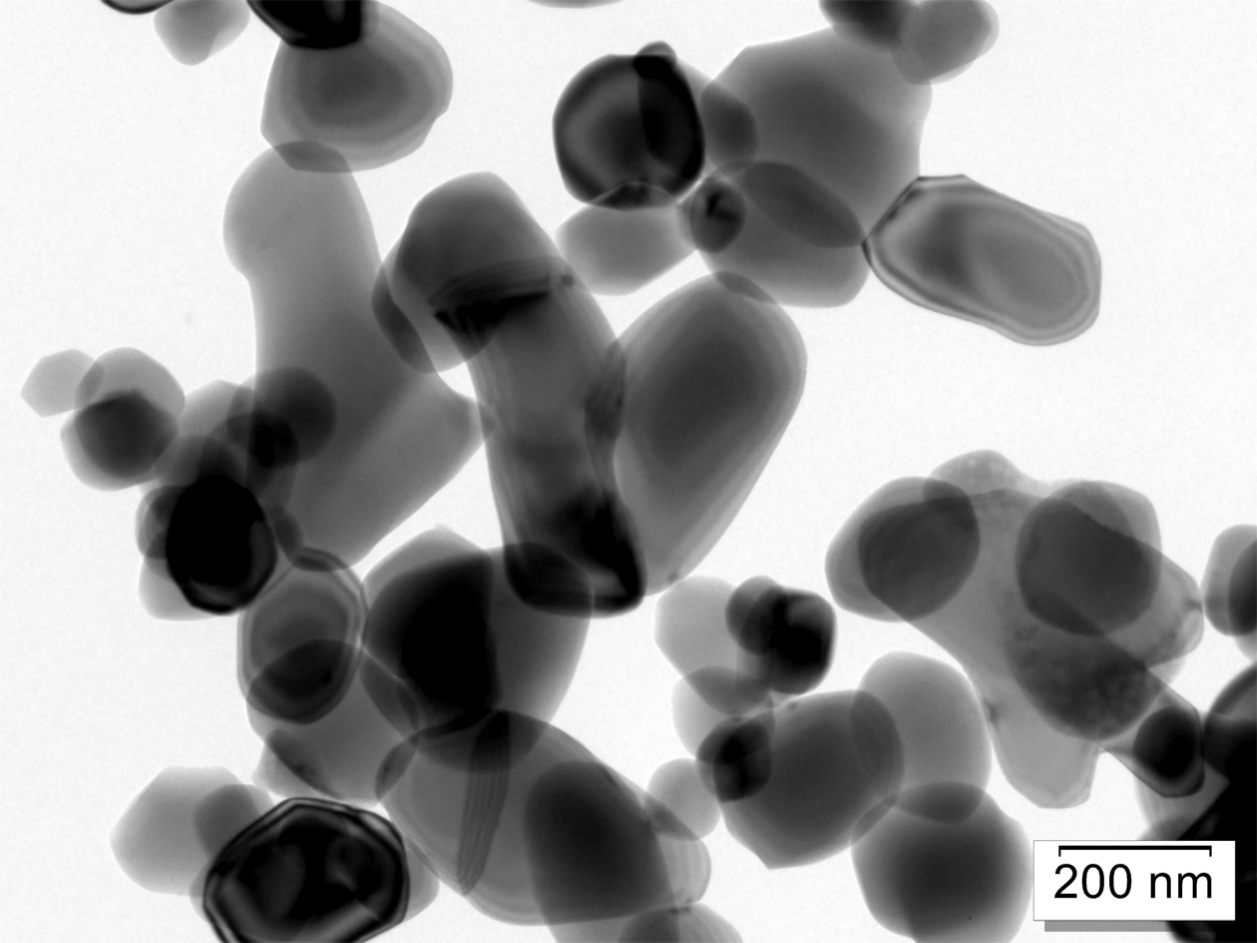
TEM image; 2048 × 2048 pixel CCD, resolution: 0.96 nm/pixel, field of view (full image): 2 μm × 2 μm.

**Figure 5:**
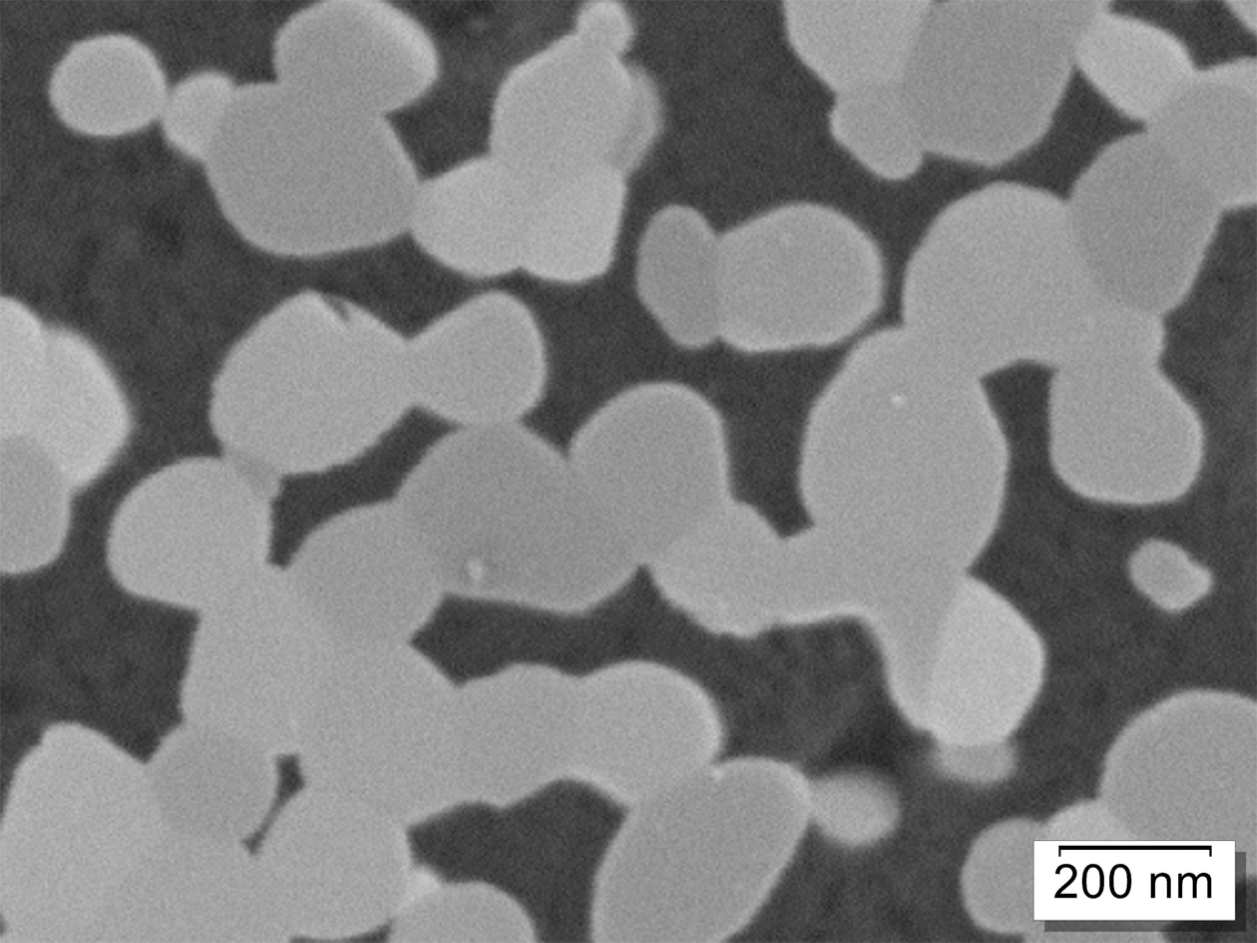
SEM cross-section image; PDBSE detector, resolution: 3.3 nm/pixel, field of view (full image): 8.5 μm × 6.4 μm.

Three of the reported measurements were made by SEM and three by TEM. The following three images (Figures 3–5) illustrate typical images used for the different methods. A top-view SEM image is shown in Figure 3 (Precheza M2, Venator M3), Figure 4 is an example of a TEM image (RCPTM, P1, P6), and Figure 5 is a cross-section SEM image (KRONOS M1).

**Figure 6:**
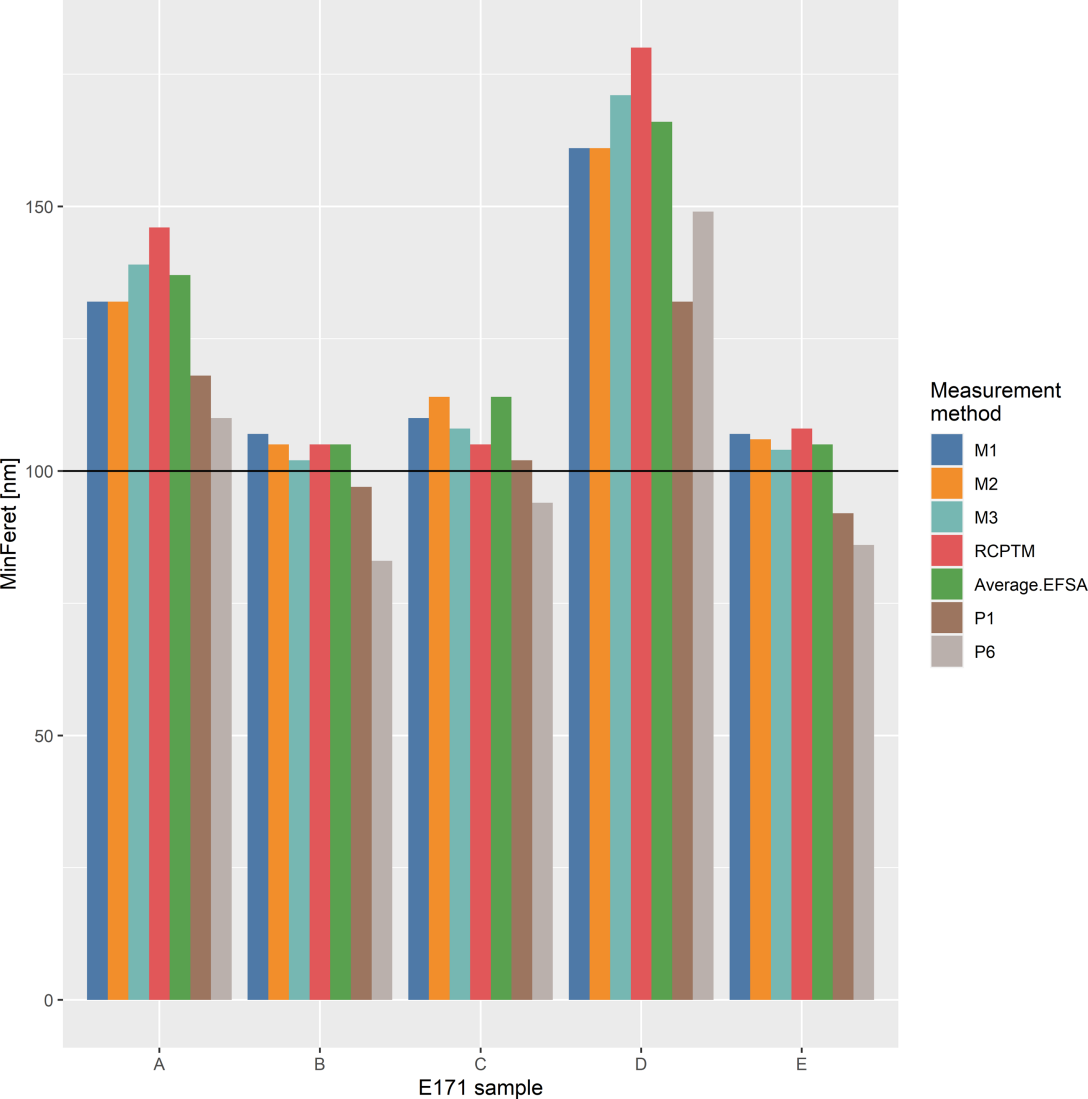
Comparison of electron microscopy results obtained by the TiO_2_ manufacturers (M1–M3) and the external lab RCPTM, and previously published results (P1, P6 [8] and average EFSA [11]).

The particle size distributions measured with each manufacturer’s method are remarkably similar, as shown in Figure 1. The D50_n_ values are close, but the tails of the distributions vary slightly, especially in the cases where a small number (less than 500) of particles are measured. This is not surprising because at less than 500 particles, the D10 or D90 tails contain only a few tens of measured particles. Hence, a few particles measured more or less in these ranges have significant influence on these parts of the curve. The largest discrepancies between the manufacturer’s measurements are observed for sample D, which has the largest primary particle size. The good agreement for the smaller samples, with all measurements coinciding within the 95% confidence interval, shows that all chosen imaging conditions are sufficient for the evaluation of E171 anatase samples. The larger deviations for sample D indicate, that the limited field of view and its implicit effect on the detection of the large particle fraction appears to be the primary cause of the deviation between the measurements shown in Figure 1. The particle size results are given in Table 6 and are visualised in Figure 6, where P1 and P6 from the publication of Verleysen et al. [8] give the smallest diameters, protocols M1, M2, and M3 provide similar results each, and RCPTM tends to obtain the largest particle size.

**Table 6:** Summary comparison of results from different methods: EFSA report [11], Verleysen et al. TEM [8], manufacturer’s SEM data, and RCPTM data.

	MinFeret (nm)						
	
	Manufacturer’s data				RCPTM	Two dispersion protocols [8]	
	
E171 sample	M1	M2	M3	average result from [11]		P1	P6

A	132	132	139	138	146	118	110
B	107	105	102	105	105	97	83
C	110	114	108	113	105	102	94
D	161	161	171	166	180	132	149
E	107	106	104	104	108	92	86

Without a certified reference material with properties similar to those of the tested samples, the accuracy of the results may be questioned, but the excellent reproducibility of the M1–M3 results is an indicator that these results are close to real values. The only difference that can be attributed to the measurements resulting in systematically different particle sizes is the sample preparation: With preparation methods P1 and P6, only a small fraction (1/200 to 1/5000) of the initially prepared 2.5 mg of sample is finally transferred to the TEM grid, whereas methods M1, M2, and M3 all bring macroscopic sample amounts to the microscope. Methods RCPTM, M2, and M3 completely rely on particle edge detection by the human eye, which is the most accurate detection method assuming good laboratory practice. Methods M1, P1, and P6 rely on automated particle detection, the former using a model-free sizing algorithm and the latter two using a sizing algorithm based on elliptical particle shape.

Based on current knowledge, sample preparation appears to be the most likely source of error, and the more dispersion and dilution steps involved, the smaller the observed particle size. This is related to the higher stability of nanoparticles in stabilised dispersions in a gravitational field.

In all cases, the median ECD calculated from the spICP-MS particle size distributions is larger than the corresponding ECD-related results of the electron microscopy measurements, which are, in turn, larger than the MinFeret-related results of the electron microscopy measurements. This is the expected outcome, since any aggregates or agglomerates remaining after a dispersion process are measured as single particle by spICP-MS, whereas EM identifies and counts the constituent primary particles. This explains why the spICP-MS particle size distributions shown in Figure 2 are multimodal and why the spICP-MS D50_n_ ECD results are much higher than equivalent D50_n_ ECD results obtained by EM. Additionally, and somewhat unexpectedly, there are nearly no particles larger than 500 nm detected in the hand-shaken sample. As a result, both instrumental limitations and fundamental constraints imply that the spICP-MS method has limited applicability for measuring primary particle sizes, especially when dealing with materials that may have a broad particle size distribution and exhibit significant agglomeration.

The EM images of the five E171 samples show that all samples are nearly spherical and have a smooth particle surface. The measured SSA is therefore a relevant parameter for direct comparison with the SSA value calculated from EM particle size distribution in the case of this material or materials with similar properties (shape and surface). Particle counting from EM micrographs is performed with only a few hundred or a few thousands of particles, whereas 1 g of titanium dioxide used in gas absorption experiments typically contains about 10^14^ particles, thus expanding the parent population and increasing statistical significance. The method for comparing the SSA with the particle size distribution from dynamic light scattering measurements [20] was adapted to the results from the electron microscopy measurements. The SSA of all five samples was calculated from the measured particle size distributions according to three different models, based on MinFeret, ECD, and using both MinFeret and MaxFeret (Cauchy). These were then compared with nitrogen absorption (BET) measurements. Surface area and volume were calculated for each measured particle and the SSA was calculated using the following formula:


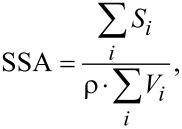


where *S_i_* is the surface area of the *i*-th particle, *V_i_* is volume of the *i*-th particle, and ρ is the material density.

**Figure 7:**
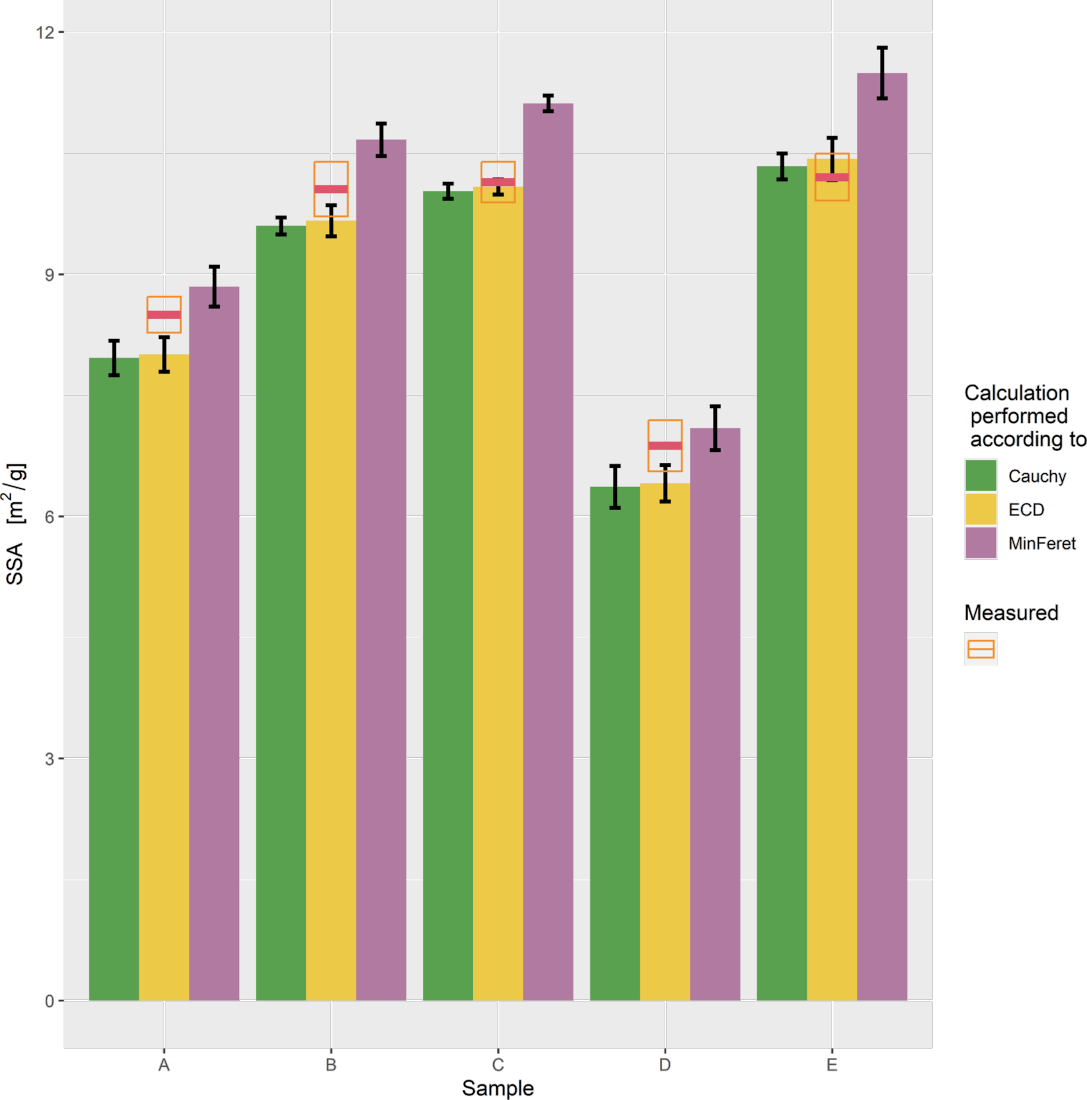
Comparison of SSA calculated from particle size distribution with SSA measured by BET.

The results presented in Table 5 and Figure 7 demonstrate that although each calculation method gives a slightly different SSA, they are very similar within a sample.

**Figure 8:**
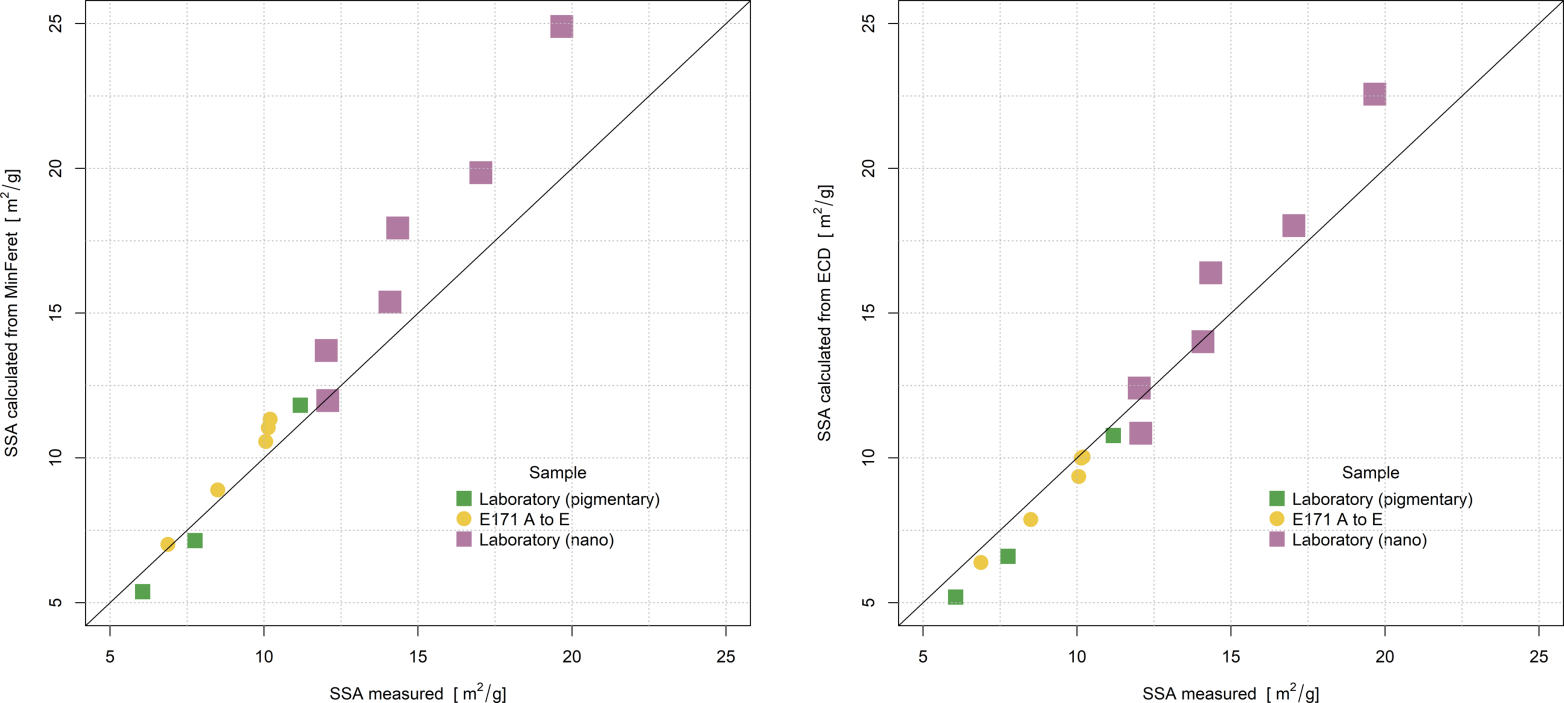
Relation of measured SSA values and SSA values calculated from EM MinFeret and ECD particle size measurements results along with the 1:1 line.

The agreement between measured and calculated values is excellent; the measured values lie somewhere between the SSA calculated using ECD and MinFeret values. Figure 8 confirms that for non-surface-treated, smooth TiO_2_ particles of samples A to E, there is good agreement between measured SSA and SSA calculated from the particle size distribution. Also, for the additional laboratory samples, the calculated SSA is valid at least in the range between 5 and 25 m^2^/g, which corresponds to a D50_n_ MinFeret of the particle size from around 50 to 250 nm for measurements by EM (method M2). The calculated SSA values are in most cases slightly larger, particularly for particles in the nanometre range, which is likely because primary particles may be partially fused within aggregates and have some inaccessible surfaces for SSA measurements.

**Figure 9:**
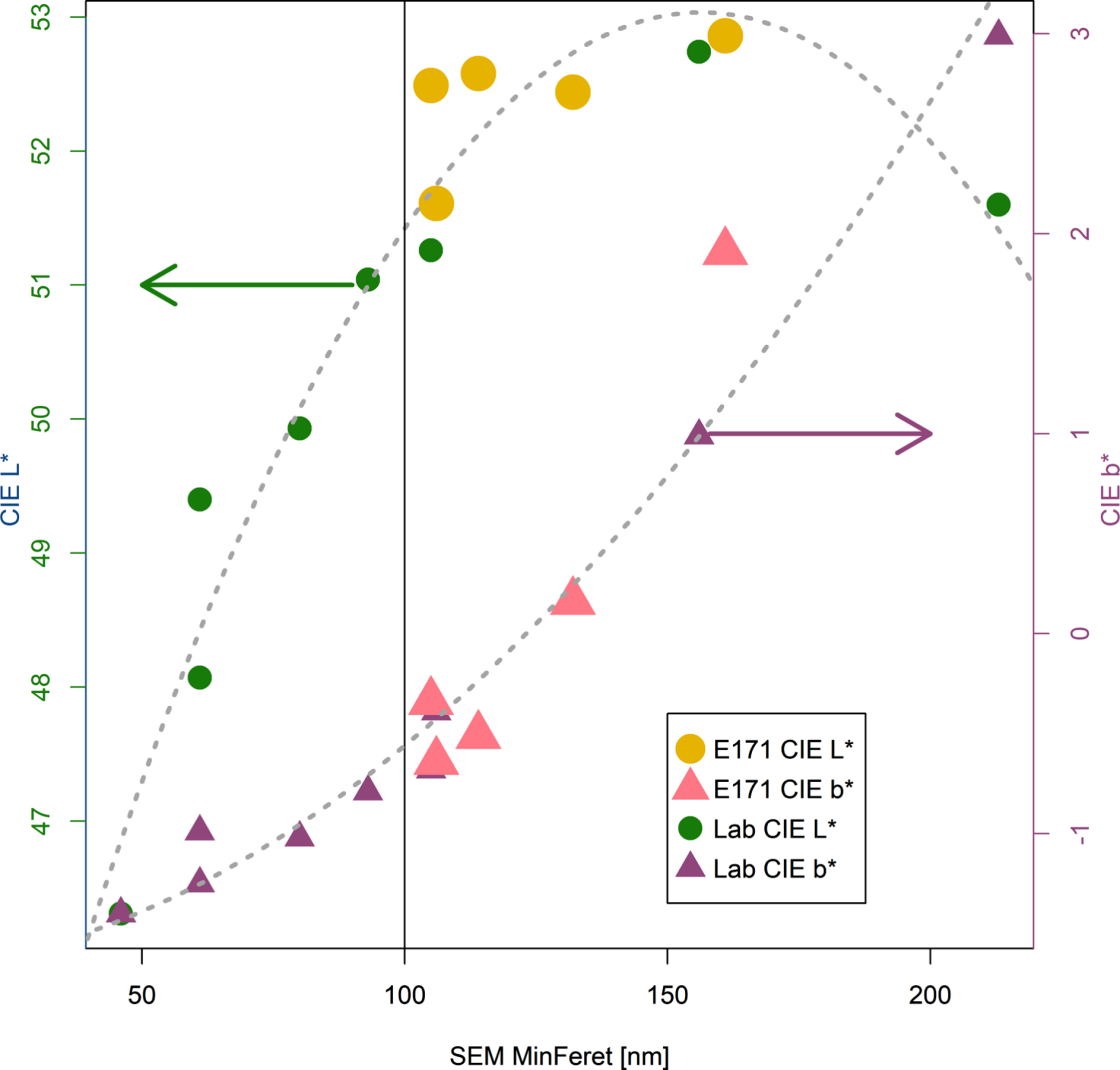
CIE L* and CIE b* values of different TiO_2_ pigment samples as a function of the D50_n_ MinFeret diameters determined by SEM.

To demonstrate the relationship between EM-based primary particle size measurements and other macroscopic measurements, we compared CIELAB colour parameters with MinFeret values for a wide range of laboratory samples and for the five samples of E171. Figure 9 shows the CIE L* (tinting strength/lightness) and the yellow tints CIE b* (tint tone/hue) of a grey paste in relation to the MinFeret particle size determined by SEM. The lightness of the pastes (tinting strength) passes through a flat maximum in the pigment MinFeret size range between 110 and 220 nm. Particles with a MinFeret well below 100 nm have limited capability to scatter visible light, resulting in poorer tinting strength, and are consequently inefficient as pigments. The CIE b* values depict the ability of the pigment to preferentially scatter longer wavelengths of the visible spectrum. The higher the CIE b* value is, the more red and green light is scattered from the paste relative to blue light. Since in an additive colour mix, red and green light together result in yellow light, a large CIE b* value is associated with a yellowish rather than a bluish colour.

To the observer, a bluish hue gives the impression of cleanliness whereas a yellowish hue is associated with an object that is somewhat old or even dirty. The optimum particle size distributions of titanium dioxide pigments are therefore a result of a balance between high CIE L* and low CIE b*. The CIE b* values are higher (more yellow) for titanium dioxide pigments with a larger mean particle size and gradually drop to lower values (more bluish tint tone) as the mean pigment particle size becomes smaller and the tinting strength decreases. This demonstrates that producing E171 in such a way that it contains a high proportion of nanoparticles is in fact undesirable from a product performance point of view as such material is an inefficient pigment with poor tinting strength.

## Conclusion

We have found that when a common set of E171 TiO_2_ pigments is independently evaluated by the laboratories of three different TiO_2_ manufacturers, three very different sample preparation and measurement procedures give similar particle size results. Given that accurate particle size measurement is a fundamental requirement for a TiO_2_ producer, it is not surprising that despite these different approaches, the results of the three laboratories all agree. Each of these manufacturers has invested a considerable amount of time and effort over several decades in order to maximise the accuracy of their particle size measurements.

The TiO_2_ pigment particles can be described as rounded polyhedra with an aspect ratio of around 1.2, which results in the smallest axis (MinFeret) being only slightly smaller than the ECD. While the EU nanomaterial definition uses the MinFeret, it is less error prone to estimate the ECD, which is also a parameter more relevant to the technical properties of TiO_2_ pigments, such as SSA and light scattering capability.

As it is not an easy task to estimate particle sizes by EM, it is useful to have an independent method to validate the primary particle size estimated by EM. While spICP-MS is widely used, especially for nanomaterials with relatively narrow particle size distributions, its use for TiO_2_ pigments, which have broad particle size distributions and are cohesive, is questionable because it cannot correctly identify and count primary particles. In addition, it provides ECD results that may not be consistent with MinFeret measurements, particularly when particles have a high aspect ratio.

In the case of cohesive materials with a broad particle size distribution, such as pigmentary TiO_2_, based on our results one would expect spICP-MS results to give larger particle sizes than EM median MinFeret measurements. When there is close agreement between the spICP-MS median particle size and the EM median MinFeret, as observed in [8], it suggests that the sample preparation procedure may not be suitable for accurate particle size measurements for such materials. A common method to provide additional confirmation of particle size is the SSA, which is a good measure for non-porous, untreated, and nearly spherical TiO_2_ particles, independent of sample dispersion. However, we advise against using average ECD or MinFeret values for such calculations. To obtain reliable and accurate results over a wide range of particle sizes, considering the full particle size distribution for SSA calculation is essential.

Simple mathematical methods can be used to calculate the SSA from the particle size distribution measured by EM, and this calculated SSA can be compared with the measured SSA. The SSA values for industrial E171 pigments range from 6 to 11 m^2^/g. For the purpose of this paper, additional laboratory samples were also evaluated with a SSA ranging from 6 to 20 m^2^/g, and the measured results confirmed this correlation. Comparison of the measured SSA with that calculated using the method described here is the most practical and reliable way of verifying EM results for nearly spherical, non-porous, and non-aggregated particles without rugged surface treatment, even if they have been prepared by different methods.

Pigmentary TiO_2_ must have a specific particle size range to scatter visible light effectively. The production process for E171 is the same as for other anatase pigments, but with greater emphasis on purity. Its particle size is designed to provide optimum light scattering in both aqueous and oil-based environments, where particle size is much more important than when the particles are surrounded by air. In the case of TiO_2_ with a refractive index of 2.5–2.9 (depending on the crystal structure), the optimum particle size for visible light scattering is around 200 nm [12]. Particles of this size scatter mostly red and green light, so the tint of such material is yellow. If a bluer shade is required, a smaller particle size will achieve this, but only down to a certain lower threshold, which is around 105–115 nm. Below this limit, the scattering power of the particles decreases significantly, and the material can no longer be used effectively as a white pigment. This behaviour was demonstrated using these laboratory samples and a grey paste evaluation, a technique commonly used in the pigment industry since the 1930s to evaluate pigment performance. The particle size of E171 TiO_2_ with an SSA of 12 m^2^/g was found to be at the upper limit for untreated anatase to be used as a pigment. Materials with a higher SSA (i.e., smaller particle size) do not scatter visible light sufficiently to be used as white pigments. This threshold corresponds approximately to a MinFeret D50_n_ particle size of 105 nm and an ECD D50_n_ particle size of 110–115 nm. By modifying the particle size (or SSA), the tint (hue) of the particles can be adjusted from blue (100–120 nm, SSA around 11 m^2^/g) through neutral (115–130 nm, 9–10 m^2^/g) to yellow (above 140 nm, 6–8 m^2^/g) according to the application requirements.

A small but significant difference between the primary particle size measurements made by the manufacturing companies and those made by the other independent scientific laboratories is noted, but the cause of the differences cannot be easily identified or resolved. We can only highlight the high level of agreement between the three TiO_2_ producer laboratories and the considerable time and resources that have gone into developing these methods, given that accurate particle size measurement is fundamental to the industry. The accuracy of these results is reinforced by the excellent agreement between the measured specific surface area results and the calculated surface area results. Whether differences in sample preparation for EM measurements are related to the discrepancies observed between the PSD results of the TiO_2_ producers and those of others can only be speculated.

The results presented here emphasise the need for a certified reference material with irregular shape, broad particle size distribution, and cohesive behaviour. Only with such a material can a meaningful comparison be made between different methods of sample preparation, measurement, and image evaluation. Reference materials for industrially important substances such as TiO_2_, SiO_2_, ZnO, and others may be advisable. Regulatory authorities or organisations such as the OECD, in collaboration with relevant industries and laboratories, should consider producing larger quantities of such reference materials and conducting extensive inter-laboratory comparisons to certify their particle size distributions.

## Experimental

### SEM investigation

The methods for sample preparation, measurement and data evaluation, including all relevant steps, are briefly described here, a more elaborate description of the cross section method (M1) can be found in literature [21]. Methods M2 and M3 used for sample preparation, image acquisition, and image processing are the same as in [11], differences in method M1 with respect to [11,21] are described below. These three methods differ substantially in all their characteristic steps, including ultrasonic bath dispersion in water, dry dispersion in a solid polymer, and the powdering onto a sticky sample stub without dispersion (Table 7). The data taken from the literature and used in the results and discussion section include probe sonication [8,11] with energies between 0.3 and 4 kJ/mL. The probe sonication in [11] was the same as described here in the method M3.

**Table 7:** EM sample preparation.

	Sample preparation

KRONOS (M1): cross section	2 g of pigment are dispersed in dry hot-mounting resin, embedded, and prepared as cross section [21].
Precheza (M2): dry	A sticky specimen stub is dusted with pigment, and excess material is blown off.
Venator (M3): sonicated dispersion	2 mg of pigment are dispersed (ultrasonication) in 50 mL of water and applied to a Si wafer. Sonication energy was 300 J/mL.

Similar operating conditions have been chosen by the three laboratories (Table 8), yet with differences in frame sizes and magnifications (Table 9). The data taken from literature and used in the Results and Discussion section were acquired using a Tecnai G2 Spirit [8], operated at 120 kV with a pixel size between 0.83 and 1.15 nm/pixel and a corresponding field of view between 3.4 µm × 3.4 µm and 4.7 µm × 4.7 µm. Further details are given in the respective references.

**Table 8:** Instruments and working conditions for EM data acquisition.

	Microscope	Acceleration Voltage	Detector	Working Distance	Size standard

KRONOS (M1)	Hitachi Regulus 8230	1 kV	PDBSE	7.5 mm	Si wafer with line structures: IMS-HR 08 3641-01 259
Precheza (M2)	Hitachi SU-70	5 kV	ETD	6.2 mm	Si wafer with etched line structures: IMS-HR 08 3641-01 259 (PTB)
Venator (M3)	Hitachi SU-6600	5 kV	ETD + InLens	5.0 mm	Si wafer with etched line structures: EM-Tec MCS-1 Magnification Calibration Std. (Micro to Nano)

**Table 9:** Image size and resolution.

	Magnification	Pixel resolution	Frame size	Number of images	Field of view

KRONOS (M1)	×12–19k	2.6-4.1 nm/pixel	2560 × 1920	>8	8.47 μm × 6.35 μm
Precheza (M2)	×70k	1.4 nm/pixel	1280 × 960	10–20	1.81 μm × 1.28 μm
Venator (M3)	×50k	2.0 nm/pixel	1280 × 960	>10	2.54 μm × 1.91 μm

Manual pigment detection has been used by two companies, which includes the user-based labelling of the particle edges of all fully visible particles in each image. Based on these boundaries, the software packages listed in Table 10 evaluate all relevant size parameters, including ECD, Feret, and shape characteristics (e.g., convexity or shape factor). The automated pigment detection used by KRONOS detects the pigments based on their grey value in the image after automatic thresholding. While the former detection methods do not require any pre-processing of the image filtering of the obtained results, the automated detection methods require both noise reduction and contrast–brightness unification prior to the automated grey-value thresholding, and a shape- and grey value-based filtering of the results to remove erroneously detected “particles”. All of these steps are defined in a workflow (macro) to ensure consistent and user-independent results [21].

**Table 10:** Image evaluation.

	Software package	Particle detection

KRONOS (M1)	Olympus, Stream	automated [21]
Precheza (M2)	Image J 1.45	manual (on touchscreen)
Venator (M3)	Media Cybernetic, ImagePro Plus 7	manual (on touchscreen)

In order to reflect the transition from a Leo 1530 VP to a Hitachi Regulus 8230 SEM, the measurement method M1 had to be adapted compared to [21] and also compared to the data published in [11]. This resulted in a change in the applied acquisition voltage and the detector used, eliminating the need for a conductive coating on the sample surface. Because of software compatibility issues with newer 64-bit operating systems, it was necessary to switch from Olympus “Analysis” to the Evident (formerly Olympus) “Stream” software package for image analysis. The changes resulted in an adjustment to the grey value filtering methodology and minor adjustments to the cutoff values for shape factor and convexity because of changes in an implemented definition. The new software package does not support the previously used watershed transform; instead, a morphological shape separator must be used. The better image quality allows one to reduce the size of the rank filter for noise reduction from five to two pixels. The differences are summarised in Table 11.

**Table 11:** Differences in M1 between [21] and [11].

Methods	As published in [21]	Current

sample	coated with 8 nm Au/Pd	no coating required
microscope	Leo 1530VP	Hitachi Regulus 8230
acceleration voltage	5 kV	2.5 kV
deceleration voltage	0 kV	1.5 kV
landing voltage	5 kV	1 kV
detector	secondary ED (ETD)	backscatter (PDBSE)
image analysis software	Olympus Analysis	Evident Stream
macro type	script	drag & drop
rank filter	5 pixels, 50%	2 pixels, 50%
shape separator	watershed transform	morphological
shape factor, limit	sphericity after Wadell, ≥0.86	circularity, ≥0.75
convexity, limit	>0.90	>0.94
grey value filter: mean: std.dev:	top 90% top 90%	μ ± 2σ (95.4%) μ ± 2σ (95.4%)

### Sample preparation and measurement protocol of RCPTM

Prior to the microscopic analyses, the supplied TiO_2_ (E171) samples were suspended in acetone (at a concentration of 6 mg/mL) and shaken in an ultrasonic bath for 10 min. Immediately after the shaking stopped, a few microliters of the fresh suspension were transferred on copper grids. The excess acetone evaporated within a few seconds, and the preparations were mounted directly to the STEM detector.

TEM analyses were performed at a voltage of 30 kV, with the beam intensity level set to 4, mostly at a magnification of 50,000× in bright-field mode. Because of the different nature of TEM micrographs, containing the aggregates of various proportions, up to nine frames were collected from the TEM measurements, and only the best were used for further particle size analyses. A TESCAN VEGA3 LMU system (S/N:VG13841481) was used, equipped with a LaB_6_ cathode, using the SE and STEM detectors, and VEGA3 control software version 4.2.26.0, build 1344.

### BET SSA measurement

The SSA measurements of the E171 samples were originally performed according to an internal procedure inspired by the ISO 9277 standard as mentioned in [11]. To directly compare the laboratory-produced samples with the E171 A–E samples, we re-measured the SSA using a similar method at Precheza, namely (i) sample degassing for 3 h at 105 °C under gaseous nitrogen (Flow Degasser, Quantachrome) and (ii) multipoint determination (5 points, NOVA 400e, Quantachrome) in the relative pressure range of 0.1–0.3 (pressure equilibration tolerance 0.1 mm Hg).

### ICP measurement method

The presented results were obtained at Agilent's Waldbronn application laboratory using an Agilent 8900 ICP-MS, in collaboration with Dr. Balski from Agilent. Sample preparation took place at KRONOS INT. Inc., Research Services, involving the dispersion of 2.5% TiO_2_ in a polyphosphate solution in water (with 2% polyphosphate for stabilisation). Dispersion was performed by an ultrasonic finger (13 mm tip) for 5 min at 250 W and 60% amplitude, equivalent to 562 J/mL. It is worth noting that usually it is not recommended to exceed sonication energies of 300 J/mL as particle size and shape of primary particles can be influenced by ultrasonic treatment [18–19].

Re-dispersion before measurement was conducted at Agilent through two methods, namely (i) hand shaking for 1 min and (ii) bath sonication for 1 min at 80 W input. Following re-dispersion at Agilent, the dilution for spICP-MS was carried out in three steps. Considering the method’s sensitivity to dilution, during the initial dilution step, the sample was kept under continuous stirring. In subsequent steps, the sample was taken manually immediately after shaking. In each instance, the sample was taken from the bottom region of the sample cuvette.

The PSD based on number was calculated from the mass spectrometer results. For each individual event (spike) detected by the mass spectrometer, an equivalent circular diameter (ECD) was calculated as the diameter of a sphere of the same mass using the literature density of the material. The number of counts for each diameter were sorted into a histogram, similar to a particle size distribution from EM. In this way a number-based particle size distribution (number of particles as function of the particle size) and the mean ECD “D50_n_ - ECD” can be calculated.

### Optical properties by grey paste measurement

A standard laboratory test to determine the optical performance of a titanium dioxide pigment is to measure the lightness L* and the yellow tint b* (yellowing) of a paste containing TiO_2_ and carbon black pigment in an oily matrix [22]. (This procedure is very close to ISO 787-24). The L* value represents the ability of a titanium dioxide pigment to efficiently scatter light, whereas the b* value indicates whether the scattered light has longer or shorter wavelength, that is, whether the scattered light is more yellow or more blue tinted. While the titanium dioxide particles scatter the light out of the mixture, the carbon black absorbs the light. The better the light scattering ability of the white pigment, the shorter the path length of the light in the paste. Thus, fewer photons are absorbed by the carbon black pigment resulting in greater brightness.

The TiO_2_ sample was mixed with carbon black, linseed oil, and filler (BaSO_4_) in defined proportions using a spatula, transferred to an automatic muller and mulled (2 × 50 revolutions). A drawdown (240 μm) of the mixture was applied on flat glass support, and the colour was evaluated by spectrophotometry (UltrascanVis, HunterLab, D65, integrating sphere, 8° geometry, 1 inch viewing port, 2 separate measurement points) and averaged. Lightness values CIE L* and yellow tints CIE b* were determined according to ISO 11664-4 and also full spectral data are recorded.

### Laboratory experimental samples preparation

Laboratory anatase samples with varying particle sizes were prepared to test the hypotheses that the optical properties of nanoscale TiO_2_ are different from pigmentary TiO_2_ and that the scattering power and blue or yellow tint (hue) are strongly dependent on primary particle size. It is relatively easy to modify particle size by calcination time and temperature [23]. TiO_2_ starting material (TiO_2_/water suspension) collected from the production site was dried (105 °C, overnight) and calcined in a laboratory calcination furnace at 750–1000 °C for 2 h. The calcined material was homogenised using a Pulverisette 2 (Fritsch) automatic mortar grinder and further ground using a Pulverisette 7 (Fritsch) planetary mill (400 rpm, 15 min) with five agate grinding balls.

### Software used for data analysis

Statistical analyses, calculations, and graphics were performed using the R software [24], the fitdistrplus package, [25] and the graphical package ggplot2 [26].

## Supporting Information

Supporting Information contains the sample data used for analysis and the code used for SSA calculations.

File 1 Additional experimental data.

## Data Availability

The data that supports the findings of this study is available from the corresponding author upon reasonable request.
