# Supplementary material for: Comparative electron microscopy particle sizing of TiO2 pigments: sample preparation and measurement
Source: Beilstein J Nanotechnol. 2024 Mar 25;15:317–32. doi: 10.3762/bjnano.15.29 (PMC10999988; doi:10.3762/bjnano.15.29)
Supplement: File 1 — Additional experimental data. [file Beilstein_J_Nanotechnol-15-317-s001.zip › support explanation.docx]

# Supplementary information

## Example of data generated by companies

Original data from each company for sample E are in Sample E.xlsx

As each company uses different way how to express results of EM measurement, it was necessary to perform standardisation for the sake of easier manipulation, statistics calculations and images – all values had to be transformed to common unit (nanometers or nanometers squared in case of area value). Example of these transformed datasets are in text files Kronos E, Venator E, Precheza E and RCPTM E. At least three columns had to be in each file, correctly named – Feret, MinFeret, Area.

## SSA calculation procedure

Vorking code example for SSA calculation from are given in file SSA calculation.r together with the file Sample A.csv on which the code can be tested.
